# Timing Matters: HIV Testing Rates in the Emergency Department

**DOI:** 10.1155/2014/575130

**Published:** 2014-09-09

**Authors:** Rebecca Schnall, Nan Liu

**Affiliations:** ^1^School of Nursing, Columbia University, New York, NY 10032, USA; ^2^Department of Health Policy and Management, Mailman School of Public Health, Columbia University, New York, NY 10032, USA

## Abstract

*Study Objectives. *In response to the 2010 New York State HIV testing law, we sought to understand the contextual factors that influence HIV testing rates in the emergency department (ED). *Methods. *We analyzed electronic health record logs from 97,655 patients seen in three EDs in New York City. We used logistic regression to assess whether time of day, day of the week, and season significantly affected HIV testing rates.* Results. *During our study period, 97,655 patients were evaluated and offered an HIV test. Of these, 7,763 (7.9%) agreed to be tested. Patients arriving between 6 a.m. and 7:59 p.m. were significantly (*P* < 0.001) more likely to be tested for HIV, followed by patients arriving between 8:00 p.m. and 9:59 p.m. (*P* < 0.01) and followed by patients arriving between 5–5:59 a.m. and 10–10:59 p.m. (*P* < 0.05) compared to patients arriving at midnight. Seasonal variation was also observed, where patients seen in July, August, and September (*P* < 0.001) were more likely to agree to be tested for HIV compared to patients seen in January, while patients seen in April and May (*P* < 0.001) were less likely to agree to be tested for HIV.* Conclusion. *Time of day and season affect HIV testing rates in the ED, along with other factors such as patient acuity and completion of other blood work during the ED visit. These findings provide useful information for improving the implementation of an HIV testing program in the ED.

## 1. Introduction

Despite the widespread availability of HIV testing, an estimated 20% of the 1.1 million human immunodeficiency virus (HIV) infected people in the USA are unaware of their infection [1]. Early diagnosis and treatment of the disease are vital to avoid increased transmission, link patients to care for treatment, and maximize therapeutic outcomes, which results in decreased morbidity and mortality and saves costs [2]. An important approach to HIV prevention is to increase HIV testing especially among those most at-risk for the disease [3]. Given the rising HIV epidemic in New York State, legislation was passed in 2010, which mandates that an HIV test be offered to all patients 13–64 years of age when they receive primary care, emergency services, or inpatient healthcare services [4].

The emergency department (ED) is an important healthcare setting for efficiently testing patients for HIV. The burden of HIV/AIDS is borne disproportionately by a growing number of racial and ethnic minorities and socioeconomically disadvantaged persons [5]. Many individuals in these groups are underinsured, have limited access to primary care, and use the ED as their sole source of medical care; therefore expanding HIV testing in the ED is especially important [6, 7]. The newest Centers for Disease Control and Prevention guidelines, which advocate routine HIV testing in all healthcare settings, emphasize the importance of EDs since they represent one of the most common sites of missed opportunity for identifying patients with unrecognized HIV infection [8]. Since the CDC guidelines have been issued, efforts have been made to introduce HIV testing into some EDs, but this has been met with mixed findings. In one study, a large portion of ED patients were willing to be screened for HIV; however, a significantly greater portion required explanation of opt-out screening [9]. In another study, nontargeted opt-out rapid HIV screening was associated with approximately 30 times the number of rapid HIV tests performed as diagnostic testing; yet only a few more patients were newly identified with HIV infection [10].

The most effective method for early identification of HIV-infected persons in the ED has yet to be determined [11]. Given the shortage of resources in the ED and the compelling evidence that the ED serves an increasingly important role in mitigating the HIV epidemic [6, 12], it is important to resolve how best to integrate routine HIV screening in the already congested ED environment [13]. Limited research has been performed to understand how the contextual factors can improve the implementation of HIV testing programs in the ED [12, 14–16].

The ED provides care 24 hours/day, 7 days/week, and the contextual factors that influence this environment are therefore dynamic. The ED environment is subject to regular variations in disease prevalence and staffing and the number of patient visits is constantly changing, which complicates planning decisions. The purpose of this study was to evaluate the relationship between HIV testing rates in the ED with time factors such as hour of day, day of week, and month. By studying these time factors, this study has the potential to elucidate the contextual factors that affect HIV testing rates in ED and thus to improve the implementation of HIV testing programs in the ED.

## 2. Methods

We secured institutional review board (IRB) approval prior to the start of our study activities. We analyzed de-identified patient data from the electronic health record (Sunrise Emergency Care, Allscripts Corporation, Chicago, IL). We only included treat-and-release patients in our study because at the time of the study the electronic alert was active only for treat-and-release patients are those who are not admitted into the hospital and directly discharged after their visits. We excluded patients over 64 years of age since they were not included in the legislation and we only assessed the adult ED since the testing process is different in the pediatric and psychiatric ED. We also excluded patients with Emergency Severity Index (ESI) 1 (*N* = 63) from our analysis because it would be irresponsible for a provider to offer an HIV test who are critically ill upon arrival to the ED and need life-saving intervention immediately. Our dataset included information on patient gender, age, race, ethnicity, and ESI [17]. Patient insurance information was not available for our analysis.

### 2.1. Study Setting

Our study included 3 adult EDs sites in New York City, which are part of the same hospital system. Two ED sites (sites 1 and 3) were part of large academic centers; the third (site 2) was associated with a community hospital. The ED volume at each of the sites ranged from 41,000 to 79,000 patients annually [18]. The three sites are all within 10 miles of each other. The prevalence of HIV in the 4 zip codes most closely bordering each hospital ranged from 16.77 to 16.79 per 1,000 persons. After the legislation, all three EDs incorporated an electronic HIV testing order set that ensures that all providers offer an HIV test to every patient treated in the ED. The order set was accompanied by an electronic hard stop alert which ensured that providers did not discharge a patient before offering an HIV test. All HIV testing at these sites was done by blood draws that were sent to a central laboratory.

### 2.2. Data Analysis

We analyzed electronic patient records from 97,655 patients seen in three EDs located in New York City. Data were managed and analyzed using R (R Foundation for Statistical Computing, Vienna, Austria). Descriptive statistics were used to characterize the patient demographics. We did not include race/ethnicity in our analysis because this information was not available for more than half of the patients. We used logistic regression to assess whether time of day, day of the week, and seasonality significantly affected HIV testing rates. Adjusted logistic regression models included the following control variables: ED site, patient age, patient gender, orders for other blood work, Emergency Severity Index (ESI), and length of stay (LOS). Patient age was included as a continuous variable. As LOS is highly skewed, we calculated the three quartiles for it and used dummy variables to represent patients in these four LOS groups. Other variables were included as categorical variables. We also conducted a regression analysis by site to see if the trends remained the same at each individual site as the overall total.

For time of day analysis, we used midnight as the reference hour. We chose midnight as the reference level because it is the natural divider of a day. We could have used other times of day as reference levels, but that would only shift the estimated log-odds ratio and would not change the main results of this paper. Similarly, we chose Monday and the month of January as a reference point for our analysis by day of week and month, respectively.

## 3. Results

From May 1, 2012, to April 30, 2013, 97,655 patients were treated in the three EDs and offered an HIV test. Our patient demographics included 43.8% male and 56.2% female. 8.1% of patients had an ESI level = 2, 49.6% of patients had an ESI level = 3, 29.5% of patients had an ESI level = 4, and 2.8% of patients had an ESI = 5. 37.3% of patients had other blood work completed during their ED visit. Mean LOS was 6.3 hours; the range is [0.017, 282.8]; the 25%, 50%, and 75% quartiles are 3.27, 5.22, and 7.97 hours, respectively (the 50% quartile is the median). The LOS of patients arriving between 6 a.m. and 7:59 p.m. is on average shorter by 0.62 hours than that of patients arriving at other times of day.

Of the 97,655 patients who were offered an HIV test, 7,758 (7.9%) agreed to be tested. Of the three variables related to timing, day of the week was not a significant predictor of HIV testing, consistently pointed out by the chi-squared test and the Wald test (*P* = 0.051). The other two timing factors (month and hour), age, sex, other blood work completed during the ED visit, ESI, LOS, and ED site were all highly significant (*P* < 0.001). Patients with an ESI = 3, 4, or 5 were more likely to agree to be tested for HIV (*P* < 0.01) as compared to patients with an ESI = 2.

Table 1 shows the impact of time of day on the rate of HIV testing controlling for other significant variables when we analyzed the full dataset. Compared to patients arriving at midnight (12:00 a.m.–12:59 a.m.), patients arriving between 6 a.m. and 7:59 p.m. were significantly (*P* < 0.001) more likely to be tested for HIV, followed by patients arriving between 8:00 p.m. and 9:59 p.m. (*P* < 0.01) and followed by patients arriving between 5–5:59 a.m. and 10–10:59 p.m. (*P* < 0.05).

When we analyzed the data by site, the effect of day of week is marginally significant (*P* = 0.03 for site 1; *P* = 0.02 for site 2; *P* = 0.003 for site 3). Both time of day and month effects are highly significant (*P* < 0.001). In particular, patients are consistently more likely to be tested during daytime hours at all sites (Table 2).

Variation by month was also observed in the full dataset, where patients seen in July, August, and September (*P* < 0.001) were more likely to agree to be tested for HIV compared to patients seen in January, while patients seen in April and May (*P* < 0.001) were less likely to agree to be tested for HIV (see Table 3).

When analyzing data by site, we found higher testing rates during the summer months in sites 1 and 3, both of which are academic medical centers. At site 2, which is a community hospital, the testing rate is consistent throughout the year (Table 4).

Older adults were less likely to be tested for HIV than younger adults (OR = 0.971; 95% CI, 0.969, 0.973; *P* < 0.001). The odds ratio 0.971 can be interpreted as the odds of getting tested will decrease by 2.9% if the patient is 1 year older, controlling for other factors. Patients who had other blood work completed during their ED visit were much more likely to be tested for HIV (OR = 2.562; 95% CI, 2.408, 2.726; *P* < 0.001). Male patients were more likely to agree to be tested than females (OR = 1.176; 95% CI, 1.120, 1.234; *P* < 0.001). Patients who had a LOS in the 2nd quartile (OR = 1.216; 95% CI, 1.128, 1.312; *P* < 0.001) and 3rd quartile (OR = 1.103; 95% CI, 1.018, 1.196; *P* < 0.05) were more likely to be tested for HIV as compared to patients in the 1st quartile of LOS. There was no significant difference in testing rates between patients in the longest LOS group as compared to those in the shortest LOS group (*P* = 0.12).

## 4. Discussion

In the USA, EDs serve as a safety net for uninsured or underinsured patients who lack a regular health care provider [19, 20]. For many of these underserved populations, the ED is their only source of healthcare services [21]. At the same time there has been an increase in demand for ED services with an insufficient supply of resources, resulting in the growing crisis of crowding [22, 23]. ED crowding has reached a “breaking point,” which greatly threatens the provision of safe and efficient care [21, 24, 25].

While the ED remains overcrowded, the integration of routine HIV testing into this oftentimes chaotic environment becomes challenging. Therefore it is not surprising that, prior to the 2006 CDC recommendation for routine HIV screening, the rate of HIV testing in US EDs was found to be only 0.3% [26]. These low testing rates in the ED are consistent across studies, even at patient visits with a blood draw when an HIV test can be easily performed. Several interventions have been developed and were found to increase HIV testing and linking patients to care. Nonetheless, these interventions have not been widely implemented suggesting existent barriers that need to be addressed to successfully implement mandatory HIV testing programs in the ED.

To complicate the integration of HIV testing into ED care, many ED providers do not think that HIV testing is in alignment with the mission of emergency medicine [27]. Public health activities such as the administration of vaccines and HIV testing are a low priority for many overburdened ED clinicians. ED providers may also be less likely to test patients for HIV if they perceive that the HIV risk of their patient is low [28, 29]. Multiple studies have shown that despite increased routine ED based testing more persons with HIV are not necessarily detected [13, 30]. Recently there has been new evidence supporting the use of targeted HIV testing especially in the ED [10, 31].

The HIV testing data in our study is unique in the fact that it was collected at a time when state legislation mandated that all providers offer patients an HIV test. The testing rate in our study is 7.9%, which is dramatically higher than national numbers where HIV testing legislation does not exist but still is suboptimal. Our study offers a unique perspective on some of the contextual factors which may be relevant for designing ED based HIV testing programs. Low HIV testing rates in the ED can be attributed to several barriers, namely, staff, space, and resources. Successful routine testing typically requires adequate staff for offering the test to a patient, performing the test, providing results, and linking patients to follow-up care [11]. Financial barriers have been cited as a key impediment to HIV testing in the ED since many providers have cited the importance of adopting rapid but relatively costly testing method so as not to increase patient LOS in the ED [32].

In our study, patients were more likely to agree to be tested during daytime hours than overnight. This pattern is consistent when we both analyzed the full dataset and also evaluated site-specific data. Patients are more likely to be tired and unwilling to agree to be tested in the middle of night. The difference in testing rates may also be the result of less clinical and ancillary staff available at night, therefore making it more unlikely for patients to agree to be tested. However, of the 7,758 patients who were tested for HIV 26 (0.34%) were found to be HIV+. Among the 26 people who had an HIV+ test, 5 were detected during 0:01 a.m. to 5:59 a.m., and the remaining 21 cases were detected between 6:00 a.m. and 19.59 p.m. Though this detection rate is not significant largely due to the fact that HIV positive is a very rare event (0.27 per 1,000 persons), this observation clearly supports the need for HIV testing during these hours.

Patients with a very short LOS were less likely to agree to be tested for HIV. These patients are usually less acute patients, who are often fast-tracked and are less likely to have other blood work completed. Our regression model supports the relationship between agreeing to an HIV test and having other blood work completed. Not surprisingly, patients with an ESI = 2 who are more critically ill would be less likely to agree to be tested for HIV. Either the provider may have deemed the patient too sick to be tested or the patient may be too focused on his acute illness to agree to be tested for HIV. It is also possible that providers may have selected the option in the Electronic Health Record stating that a patient declined to be tested because they deemed a patient too ill to decide about HIV testing during the visit. Women were less likely to agree to be tested for HIV which is consistent with findings from other studies [33, 34].

Seasonal variation was also observed, where patients seen in July, August, and September were more likely to agree to be tested for HIV compared to patients seen in January, in particular in those academic medical centers. Our data did not show a large difference in number of patient visits during these months (see Table 3). There has been limited and conflicting research on patient crowding by season. In one study, July was the busiest month of the year [35] and in another study July and November were the least busy months [36]. In the summer months, providers may be more likely to encourage patients to be tested since new residents begin work in July and they have recently been exposed to in-depth orientation sessions and so have a heightened awareness of the need to order an HIV test for their patients [37]. However, given that this effect is not lasting, it suggests that hospital administration needs to exert continuous effort in improving HIV testing in EDs in order to preserve consistent performances.

Findings from our study provide some insight into how best to allocate additional staff and resources to increase HIV testing rates in the ED. Past research has assessed provider factors which may influence testing practices, such as appropriate staffing and funding [38]. Our results support these provider-related recommendations since there are significant effects of time and seasonality on HIV testing rates, which may be correlated with variation in staffing and resource allocation in the ED.

*Limitations*. The single geographic area limits the generalizability of our findings. This study was conducted in 3 busy urban EDs, which may vary greatly from rural and less congested EDs. In addition, the electronic data which we analyzed was self-reported by the providers. One of the major potential limitations of this work is the lack of information on how many providers documented an offer of the test but did not truly offer the HIV test to their patients. It is possible that a provider may not have offered the test but documented having done so in order to avoid the electronic alert and be able to discharge a patient without delay.

## 5. Conclusions

Time of day and season affect HIV testing rates in the ED, along with other factors such as patient acuity, LOS, and completion of other blood work during the ED visit. Further research should investigate additional factors such as staffing, laboratory capacity, and patient fatigue to maximize the utility of HIV testing programs in the ED.

## Figures and Tables

**Table 1:** Adjusted odds ratio of HIV testing by hour of patient admission to the ED.

Hour	Number of patients	Adjusted odds ratio	95% confidence interval	*P* value
12:00 a.m. (reference)	2,835	1.00		N.A.
1:00 a.m.	2,447	1.20	0.95, 1.52	N.S.
2:00 a.m.	2,042	1.04	0.80, 1.34	
3:00 a.m.	1,790	0.85	0.64, 1.13	
4:00 a.m.	1,618	1.20	0.92, 1.56	

5:00 a.m.	1,718	1.35	1.05, 1.74	<0.05

6:00 a.m.	1,767	2.02	1.60, 2.54	<0.001
7:00 a.m.	2,628	2.08	1.68, 2.58	
8:00 a.m.	4,120	2.42	1.98, 2.95	
9:00 a.m.	5,505	2.58	2.13, 3.11	
10:00 a.m.	6,089	2.10	1.73, 2.54	
11:00 a.m.	6,101	2.23	1.84, 2.70	
12:00 p.m.	5,904	2.16	1.78, 2.62	
1:00 p.m.	5,769	1.96	1.61, 2.38	
2:00 p.m.	5,636	1.77	1.46, 2.16	
3:00 p.m.	5,580	1.91	1.57, 2.32	
4:00 p.m.	5,385	1.86	1.53, 2.27	
5:00 p.m.	5,169	1.67	1.37, 2.04	
6:00 p.m.	4,996	1.58	1.29, 1.93	
7:00 p.m.	4,752	1.46	1.19, 1.79	

8:00 p.m.	4,489	1.36	1.10, 1.67	<0.01
9:00 p.m.	4,180	1.32	1.07, 1.64	

10:00 p.m.	3,670	1.29	1.04, 1.60	<0.05

11:00 p.m.	3,467	1.20	0.96, 1.50	N.S.

**Table 2:** Adjusted odds ratio of HIV testing by hour of patient arrival in the ED categorized by site.

	Reference time	Higher chance (*P* < 0.05)	The same chance (*P* ≥ 0.05)	Lower chance (*P* < 0.05)	Total patient visits
Site 1	12:00 a.m.–0:59 a.m.	6:00 a.m.–6:59 a.m.; 9:00 a.m.–9:59 a.m.; 11:00 a.m.–00:59 p.m.; 3:00 p.m.–4:59 p.m.; 6:00 p.m.–8:59 p.m.; 10:00 p.m.–10:59 p.m.	Other times		34237

Site 2	12:00 a.m.–0:59 a.m.	8:00 a.m.–9:59 a.m.; 11:00 a.m.–11:59 a.m.;	Other times	6:00 p.m.–6:59 p.m.; 8:00 p.m.–9:59 p.m.;	21701

Site 3	12:00 a.m.–0:59 a.m.	1:00 a.m.–1:59 a.m.; 5:00 a.m.–9:59 a.m.; 11:00 p.m.–11:59 p.m.	Other times		41717

Overall	12:00 a.m.–0:59 a.m.	5:00 a.m.–10:59 p.m.	Other times		97655

**Table 3:** Impact of month of patient admission to the ED on the odds ratios of HIV testing.

Hour	Number of patients visits	Adjusted odds ratio	95% confidence interval	*P* value
January (reference)	8,597	1		N.A.

February	8,197	0.91	0.81, 1.03	N.S.
March	8,623	0.90	0.80, 1.01	

April	8,300	0.78	0.69, 0.88	<0.001
May	7,524	0.80	0.71, 0.91	

June	8,444	1.05	0.93, 1.18	N.S.

July	7,819	1.49	1.34, 1.66	<0.001
August	8,202	1.29	1.15, 1.43	
September	8,138	1.22	1.09, 1.36	

October	7,981	1.10	0.98, 1.23	N.S.
November	7,749	0.94	0.84, 1.06	
December	8,081	1.07	0.96, 1.20	

**Table 4:** Adjusted odds ratio of HIV testing by month of patient arrival in the ED categorized by site.

	Number of patient visits	Reference month	Higher chance (*P* < 0.05)	The same chance (*P* ≥ 0.05)	Lower chance (*P* < 0.05)
Site 1	34237	January	May, June, July, August, October	February, March, April, September, November, December	
Site 2	21701	January		February, March, April, June, July, August, September, October, November, December	May
Site 3	41717	January	July, August, September	February, June, October, December	March, April, May, November

Overall	97655	January	July, August, September	February, March, June, October, November, December	April, May
